# Health-Related Quality of Life Among US Veterans and Civilians by Race and Ethnicity

**DOI:** 10.5888/pcd9.110138

**Published:** 2012-05-31

**Authors:** Cecily Luncheon, Matthew Zack

**Affiliations:** Author Affiliations: Cecily Luncheon, Centers for Disease Control and Prevention, Atlanta, Georgia.

## Abstract

**Introduction:**

Among veterans, having been selected into the military and having easy access to medical care during and after military service may reduce premature mortality but not morbidity from mental distress and may not improve health-related quality of life. The objective of this study was to determine whether veterans in different racial/ethnic groups differ in their health-related quality of life from each other and from their civilian counterparts.

**Methods:**

Among 800,000 respondents to the 2007–2009 Behavioral Risk Factor Surveillance System surveys, approximately 110,000 identified themselves as veterans and answered questions about their sociodemographic characteristics, self-rated health, and recent health-related quality of life. Nonoverlapping 95% confidence intervals of means distinguished veterans and civilians of different racial/ethnic groups.

**Results:**

Veteran and civilian American Indians/Alaska Natives reported more physically unhealthy days, mentally unhealthy days, and recent activity limitation days than their veteran and civilian counterparts in other racial/ethnic groups. Non-Hispanic white veterans and Hispanic veterans reported more physically unhealthy days, mentally unhealthy days, and recent activity limitation days than their civilian counterparts.

**Conclusion:**

Unlike findings in other studies, our findings show that veterans’ health-related quality of life differs from that of civilians both within the same racial/ethnic group and among different racial/ethnic groups. Because once-healthy soldiers may not be as healthy when they return to civilian life, assessing their health-related quality of life over time may identify those who need help to regain their health.

## Introduction

Each soldier’s experience in the military is unique, whether the soldier volunteered or was drafted into military service (1). After being selected, completing basic training, and going off to their assignments, all soldiers have the common experience that they are generally healthier than those excluded from military service (2). Preliminary screening disqualifies those who are less physically and psychologically fit, remaining in the service requires meeting physical and psychological standards, and accessing medical care is easier during and after military service. This “healthy soldier” effect may reduce premature mortality among soldiers compared with their nonsoldier peers even after military service has ended.

This benefit of reduced premature mortality for soldiers may not carry over to reduced morbidity from mental distress and improved health-related quality of life (HRQOL) (3-5). Overall quality of life involves individual and subjective evaluations of the positive and negative aspects of life based on one’s values and culture and includes who one is (part of a family, health, function), what one does (cares for others, works, goes to school), and where one lives (community, nation) (6). HRQOL is that part of overall quality of life that affects physical and mental health (7,8). HRQOL includes a person’s perceptions of his or her physical and mental health, which results from health risks, conditions, functional status, socioeconomic status, and social support. For example, HRQOL in the general US population varies by sociodemographic characteristics including race/ethnicity, risky behaviors, reported chronic health conditions, activity limitation, and social support (9-19).

Previous studies have examined the HRQOL of veterans with mixed results (20-23). Some studies compared HRQOL among active duty, reserve, and veteran military personnel with that of those with no military service without directly analyzing HRQOL by race/ethnicity (20-22). Another study compared scores on the Medical Outcomes Study Short Form 36-item Survey for Veterans for active duty and Reserve/National Guard military personnel by race/ethnicity to US normative scale scores (23). However, none of these studies has analyzed racial/ethnic differences in HRQOL of representative samples of veterans and their nonveteran civilian counterparts. The objective of this study was to determine whether veterans in different racial/ethnic groups differ in their HRQOL from each other (primary) and from their civilian counterparts (secondary).

## Methods

This study is a descriptive analysis of cross-sectional data from respondents to the 2007–2009 surveys of the Behavioral Risk Factor Surveillance System (BRFSS). The BRFSS is an annual random-digit–dialed telephone survey in all 50 US states, the District of Columbia, Puerto Rico, the Virgin Islands, and the US Pacific territories (24). Eligible participants are adults (1 per household) aged 18 years or older interviewed about their health status, access to health care, and health behaviors. The Centers for Disease Control and Prevention (CDC) institutional review board has reviewed and approved the BRFSS protocol. The BRFSS method, design, questionnaires, and data sets are available in the public domain (24).

### Sample

Of 1,278,028 participants in the 2007–2009 BRFSS, 801,862 (63%) answered a question about their status as a veteran (see definition below) and identified themselves as either non-Hispanic whites, non-Hispanic blacks, American Indians/Alaska Natives, or Hispanics. Twelve percent (n = 110,365) of these reported being a veteran, 100,829 (92%) men and 9,536 (8%) women (values are weighted). We compared veterans and their civilian counterparts within racial/ethnic groups by age, marital status, educational level, employment status, annual income, and HRQOL.

### Measures

The HRQOL items used for this study were self-rated health (excellent, very good, good, fair, or poor), physically unhealthy days (the number of days during the past 30 days when one’s physical health was not good), mentally unhealthy days (the number of days during the past 30 days when one’s mental health was not good), and recent activity limitation days (the number of days during the past 30 days when one’s physical or mental health kept one from doing one’s usual activities). The question about veteran status remained the same during the 2007–2009 BRFSS surveys: “Have you ever served on active duty in the United States Armed Forces, either in the regular military or in a National Guard or military reserve unit? Active duty does not include training for the Reserves or National Guard, but DOES include activation, for example, for the Persian Gulf War.” However, the response choices differed in the 2009 questionnaires from those in the 2008 and 2007 questionnaires. In 2009, participants chose from 7 responses: 1) yes, now on active duty; 2) yes, on active duty during the last 12 months, but not now; 3) yes, on active duty in the past, but not during the last 12 months; 4) no, training for Reserves or National Guard only; 5) no, never served in the military; 6) don’t know/not sure; and 7) refused. In the 2007 and 2008 BRFSS, there were 4 choices: yes, no, don’t know/not sure, and refused. For this study, we defined veterans as those answering yes to these questions on any of the 3 surveys and civilians as those answering no to these questions. We excluded from the analysis those answering don’t know/not sure and those refusing to answer these questions.

The demographic characteristics analyzed were the following: race/ethnicity (non-Hispanic white, non-Hispanic black, American Indian/Alaska Native, or Hispanic); age group (18–24, 25–34, 35–44, 45–54, 55–64, or ≥65 y), marital status (currently married or not), educational level (≤high school, attended college or technical school, or graduated from college or technical school), employment status (currently employed for wages or self-employed, not currently employed [includes the unemployed, students, homemakers, or unable to work], or retired), and annual household income (<$15,000, $15,000–$24,999, $25,000–$34,999, $35,000–$49,999, or ≥$50,000).

### Statistical analysis

To account for the BRFSS complex sample design and sampling weights, we used SAS-callable SUDAAN version 9.2 (RTI International, Research Triangle Park, North Carolina) to estimate demographic characteristics and self-rated health and mean unhealthy days by veteran status and race/ethnicity, both unadjusted and adjusted for sex, age group, marital status, educational level, employment status, and annual household income. Nonoverlapping 95% confidence intervals of means statistically distinguished veterans and civilians of different racial/ethnic groups.

## Results

Women were more likely than men to be civilians, although non-Hispanic black and Hispanic women were more likely than non-Hispanic white women to be veterans (Table 1). Hispanic veterans usually reported their health as being better than that of their civilian counterparts, non-Hispanic blacks and American Indian/Alaska Native veterans as about the same, and non-Hispanic white veterans as being worse; non-Hispanic white civilians generally reported their health as better than that of civilians in other racial/ethnic groups.

**Table 1:** Characteristics of Veterans and Civilians by Race/Ethnicity, Behavioral Risk Factor Surveillance System, 2007–2009^a^

**Characteristics**	**Veterans (n = 110,365)**				**Civilians (n = 691,497)**			

	Non-Hispanic White	Non-Hispanic Black	American Indian/ Alaska Native	Hispanic	Non-Hispanic White	Non-Hispanic Black	American Indian/ Alaska Native	Hispanic

	% (95% CI)															
**Female**	7 (6-8)	12 (11–14)	9 (6-13)	10 (8-13)	59 (58–60)	60 (59–62)	54 (51–57)	52 (50–53)
**Age group, y**																
18–24	2 (1-3)	3 (2-5)	4 (1-9)	6 (4-9)	11 (10–12)	14 (13–16)	19 (16–23)	19 (17–20)
25–34	7 (6-8)	12 (10–15)	8 (4-14)	18 (15–22)	17 (17–18)	21 (20–22)	20 (18–23)	27 (26–28)
35–44	12 (11–13)	23 (20–26)	15 (11–20)	18 (15–21)	20 (19–20)	21 (20–22)	19 (17–21)	23 (22–24)
45–54	14 (13–14)	23 (20–25)	19 (15–24)	16 (14–19)	21 (20–22)	19 (17–20)	19 (17–21)	15 (14–16)
55–64	24 (23–25)	19 (17–21)	27 (22–32)	17 (15–20)	15 (14–15)	13 (12–14)	13 (11–14)	9 (8-10)
≥65	41 (40–42)	20 (18–22)	27 (22–33)	25 (22–28)	16 (16–17)	12 (11–13)	10 (9-12)	8 (7-9)
**Married**	75 (74–76)	59 (57–62)	61 (54–67)	69 (65–72)	63 (62–64)	37 (36–39)	47 (44–50)	54 (52–55)
**Education**																
High school diploma or less	34 (33–35)	35 (32–37)	38 (33–44)	35 (31–38)	36 (35–36)	49 (48–50)	55 (53–58)	64 (63–65)
Some college or technical school	30 (29–31)	38 (35–40)	34 (28–40)	37 (33–40)	27 (26–28)	27 (26–29)	27 (24–30)	20 (19–21)
Graduated from college or technical school	37 (35–38)	28 (25–30)	28 (22–34)	29 (25–32)	38 (37–38)	24 (23–25)	18 (16–20)	16 (15–17)
Employment																
Employed	49 (48–50)	57 (55–60)	49 (43–55)	60 (56–63)	63 (62–63)	58 (56–59)	55 (52–57)	61 (59–62)
Not employed	9 (8-10)	18 (15–20)	17 (13–22)	14 (11–17)	22 (22–23)	30 (29–32)	35 (32–37)	33 (32–35)
Retired	42 (41–43)	25 (23–27)	34 (29–40)	27 (24–30)	15 (14–16)	12 (11–13)	11 (9-12)	6 (5-7)
**Annual household income, $**																
<15,000	5 (4-5)	9 (7-11)	13 (9-17)	10 (8-13)	7 (6-7)	17 (16–18)	18 (15–21)	22 (21–23)
15,000–24,999	13 (12–14)	15 (13–17)	19 (14–24)	15 (12–18)	12 (11–13)	23 (22–24)	24 (21–26)	28 (26–29)
25,000–34,999	12 (11–13)	13 (11–15)	16 (11–21)	12 (10–15)	10 (9-11)	15 (14–16)	14 (12–16)	15 (13–16)
35,000–49,999	18 (17–19)	20 (18–23)	17 (12–22)	17 (14–20)	15 (14–15)	15 (14–16)	15 (12–17)	13 (12–14)
≥50,000	53 (52–54)	43 (40–46)	36 (30–43)	46 (42–50)	57 (56–57)	30 (29–32)	30 (28–33)	22 (21–24)
**Self-rated health**																
Excellent	18 (17–19)	18 (16–21)	16 (12–21)	22 (19–26)	22 (22–23)	17 (16–18)	17 (15–20)	17 (15–18)
Very good	32 (31–33)	27 (24–29)	23 (18–28)	28 (24–31)	37 (36–38)	26 (25–28)	25 (22–28)	20 (19–22)
Good	31 (30–32)	34 (31–36)	34 (28–40)	30 (27–33)	28 (27–29)	36 (34–37)	34 (31–37)	37 (35–38)
Fair	13 (12–14)	15 (13–17)	15 (11–19)	14 (12–17)	9 (9-10)	16 (14–17)	15 (13–17)	22 (20–23))
Poor	6 (5-7)	6 (4-8)	13 (9-17)	6 (5-9)	4 (3-4)	6 (5-6)	9 (7-10)	5 (4-6)

Abbreviation: CI, confidence interval.

^a^ Percentages and their 95% confidence intervals (CIs) are based on a weighted analysis to account for the survey’s complex sample design.

American Indian/Alaska Native veterans reported more physically unhealthy days and recent activity limitation days than veterans in other racial/ethnic groups (Table 2). American Indian/Alaska Native civilians said they had more physically unhealthy days, mentally unhealthy days, and recent activity limitation days than civilians in other racial/ethnic groups. American Indian/Alaska Native veterans and non-Hispanic white veterans described themselves as having more physically unhealthy days and non-Hispanic white veterans reported more recent activity limitation days than their civilian counterparts. Non-Hispanic white and black veterans reported fewer mentally unhealthy days than their civilian counterparts.

**Table 2:** Unadjusted^a^ and Adjusted^b^ Mean Unhealthy Days for Veterans and Civilians by Race/Ethnicity, Behavioral Risk Factor Surveillance System, 2007–2009

**Measure**	**Non-Hispanic White**		**Non-Hispanic Black**		**American Indian/Alaska Native**		**Hispanic**	

	Veterans	Civilians	Veterans	Civilians	Veterans	Civilians	Veterans	Civilians

	Mean (95% CI)							
**Physically unhealthy days**								
Unadjusted	4.2 (4.1–4.4)	3.4 (3.4–3.5)	4.4 (3.9–4.9)	3.9 (3.7–4.0)	7.3 (5.9–8.6)	5.3 (4.9–5.7)	4.2 (3.6–4.7)	3.6 (3.5–3.8)
Adjusted	4.1 (3.9–4.2)	3.7 (3.6–3.8)	4.0 (3.5-4.5)	3.2 (3.0–3.3)	6.7 (5.3–8.1)	4.7 (4.2–5.1)	4.1 (3.5–4.7)	2.8 (2.6–3.0)
**Mentally unhealthy days**								
Unadjusted	2.6 (2.4–2.7)	3.4 (3.3–3.5)	3.3 (2.9–3.7)	4.0 (3.8–4.1)	3.8 (2.9–4.7)	5.1 (4.7–5.6)	3.0 (2.6–3.4)	3.6 (3.4–3.7)
Adjusted	4.0 (3.8–4.1)	3.6 (3.5–3.7)	3.6 (3.2–4.1)	3.1 (2.9–3.3)	4.2 (3.3–5.1)	4.3 (3.8–4.7)	3.7 (3.2–4.2)	2.4 (2.2–2.6)
**Recent activity limitation days**								
Unadjusted	2.5 (2.4–2.6)	2.1 (2.0–2.2)	2.9 (2.4–3.3)	2.6 (2.4–2.7)	4.7 (3.6–5.8)	3.9 (3.5–4.3)	2.6 (2.1–3.1)	2.1 (2.0–2.3)
Adjusted	2.6 (2.5–2.8)	2.3 (2.2–2.4)	2.6 (2.1–3.0)	2.0 (1.8–2.1)	4.4 (3.2–5.5)	3.3 (2.9–3.7)	2.6 (2.1–3.1)	1.4 (1.2–1.5)

Abbreviation: CI, confidence interval.

^a^ Means and their 95% confidence intervals (CIs) are based on a weighted analysis to account for the survey’s complex sample design.

^b^ Means and their 95% CIs are weighted to account for the survey’s complex sample design and adjusted for sex, age group, marital status, educational level, employment status, and annual household income.

After adjusting for sex, age, marital status, educational level, employment status, and annual household income, American Indian/Alaska Native veterans still reported more physically unhealthy days and recent activity limitation days than veterans in other racial/ethnic groups (Table 2). American Indian/Alaska Native civilians still said they had more physically unhealthy days, mentally unhealthy days, and recent activity limitation days than civilians in other racial/ethnic groups. Veterans in all racial/ethnic groups reported more physically unhealthy days than their civilian counterparts, but only non-Hispanic white and Hispanic veterans said they had more mentally unhealthy days and recent activity limitation days than their civilian counterparts.

## Discussion

This study explored differences in the associations between the HRQOL of veterans and civilians by racial/ethnic group. Despite the “healthy soldier” effect, other studies have documented poorer mental and physical health in some veterans, which might be expected to affect their health perceptions or HRQOL (3-5,25-27). Yet, in none of these studies was race or ethnicity associated with poorer mental and physical health after accounting for other potentially confounding variables. In this study, however, the HRQOL of veterans differed from that of their civilian counterparts both within the same racial/ethnic group and among different racial/ethnic groups. What happened to these veterans during or after military service may have affected these differences in their current HRQOL. The higher number of mean physically unhealthy days among veterans of all racial/ethnic groups compared with that of their civilian counterparts, even after adjustment, may indicate persistent effects of physical trauma associated with military service (28). Veterans who belonged to racial/ethnic groups that may be discriminated against more often, American Indians/Alaska Natives and non-Hispanic blacks, did not differ from their civilian counterparts with respect to their mental or activity-limiting HRQOL, perhaps because discrimination against these groups after military service affects these aspects of HRQOL more than military service alone (29,30). However, veterans who belong to racial/ethnic groups that may be less discriminated against, non-Hispanic whites and Hispanics, still reported worse mental and activity-limiting HRQOL than their civilian counterparts, suggesting that military service can affect these aspects of HRQOL.

Compared with these other studies, our study had several strengths. It analyzed HRQOL in different racial/ethnic groups and had sizable numbers of respondents in these groups, allowing for the adjustment of potentially confounding sociodemographic characteristics. The BRFSS questions on HRQOL have acceptable validity and reliability (7,9). Because these HRQOL questions preceded those asking about veterans status, the ascertainment of status as a veteran probably did not affect responses about HRQOL.

This study also had several limitations. Because BRFSS depends on self-reported experiences, we could not corroborate reports of veteran status, although respondents would not benefit from falsely reporting themselves as veterans or denying they were veterans. Moreover, HRQOL is inherently subjective, and we could not corroborate differences in HRQOL with objective indicators of health and functional status (eg, physician records of diagnosed disease, hospitalizations) that affect HRQOL. Because the questions in the 2007–2009 BRFSS do not distinguish between participation in the military and exposure to combat and do not ask about duration of military service (www.cdc.gov/brfss/), we could not tell whether exposure to combat and duration of military service affected the observed differences in HRQOL among the different racial/ethnic groups of veterans. Moreover, because BRFSS is cross-sectional, we could not tell whether the observed differences in HRQOL between veterans and civilians occurred because of events during military service or afterward. The small number of women veterans in some racial/ethnic groups precluded comparison of their HRQOL with that of their civilian counterparts. Until recently, BRFSS has been based on landline residential telephones and excludes US adults who use only cell phones and whose sociodemographic characteristics and responses to BRFSS may differ from those who use landline residential telephones.

The HRQOL differences in this study between veterans and their civilian counterparts and among veterans in different racial/ethnic groups may indicate persistent health problems associated with military service, persistent discrimination against certain racial/ethnic groups despite their military service, or both. Because once-healthy soldiers may not be as healthy when they return to civilian life, assessing their HRQOL over time may identify those who need help to regain their health.

## Acknowledgments

This project was supported in part by appointment to the Research Participation Program for CDC administered by the Oak Ridge Institute for Science and Education through an agreement between the Department of Energy and CDC. This research received no specific grant from any funding agency in the public, commercial, or nonprofit sectors.
